# Metformin coordinates osteoblast/osteoclast differentiation associated with ischemic osteonecrosis

**DOI:** 10.18632/aging.102796

**Published:** 2020-02-11

**Authors:** See-Hyoung Park, Mi-Ae Kang, Young Jae Moon, Kyu Yun Jang, Jung Ryul Kim

**Affiliations:** 1 Department of Bio and Chemical Engineering, Hongik University, Sejong, Korea; 2 Department of Biological Science, Gachon University, Seongnam, Korea; 3 Department of Orthopaedic Surgery, Chonbuk National University Medical School, Research Institute of Clinical Medicine of Chonbuk National University-Biomedical Research Institute of Chonbuk National University Hospital and Research Institute for Endocrine Sciences, Jeonju, Korea; 4 Department of Pathology, Chonbuk National University Medical School, Research Institute of Clinical Medicine of Chonbuk National University-Biomedical Research Institute of Chonbuk National University Hospital and Research Institute for Endocrine Sciences, Jeonju, Korea

**Keywords:** metformin, angiopoietin 1, ischemic osteonecrosis, osteoblastic differentiation

## Abstract

In this study, we aimed to identify a candidate drug that can activate endogenous Angiopoietin 1 (Ang1) expression via drug repositioning as a pharmacological treatment for avascular osteonecrosis. After incubation with 821 drugs from the Food and Drug Administration (FDA)-approved drug library, Ang1 expression in U2OS cell culture media was examined by ELISA. Metformin, the first-line medication for treatment of type 2 diabetes, was selected as a candidate for in vitro and in vivo experimental evaluation. Ang1 was induced, and alkaline phosphatase activity was increased by metformin treatment in U2OS and MG63 cells. Wound healing and migration assay showed increased osteoblastic cell mobility by metformin treatment in U2OS and MG63 cells. Metformin upregulated expression of protein markers for osteoblastic differentiation in U2OS and MG63 cells but inhibited osteoclastic differentiation in Raw264.7 cells. Metformin (25 mg/kg) protected against ischemic necrosis in the epiphysis of the rat femoral head by maintaining osteoblast/osteocyte function and vascular density but inhibiting osteoclast activity in the necrotic femoral head. These findings provide novel insight into the specific biomarkers that are targeted and regulated by metformin in osteoblast differentiation and contribute to understanding the effects of these FDA-approved small-molecule drugs as novel therapeutics for ischemic osteonecrosis.

## INTRODUCTION

Ischemic necrosis of the femoral head (INFH) can lead to permanent femoral head deformity, severely compromised hip joint longevity, and premature end-stage osteoarthritis as early as the third decade of life. Use of biological agents to preserve the femoral head and avoid joint replacement surgery is currently under investigation. The therapeutic concepts of angiogenesis and secondary osteogenesis have gained considerable attention in terms of preventing development of femoral head deformities after ischemic osteonecrosis [1].

Thus far, several kinds of growth factors have been reported to regulate angiogenesis [2]. Among them, Angiopoietin 1 (Ang1) is a well-known angiogenic factor that plays a pivotal role in stimulating and developing new vasculature for bone formation [3, 4]. Previously, we reported that a recombinant COMP-Ang1 protein, a chimeric form of Ang1, facilitates necrotic femoral head repair via enhancement of angiogenesis [5].

It is both expensive and time-consuming to develop new drugs [6]. Recently, a novel concept called “Drug Repositioning or Repurposing” has been introduced to find and develop new drugs [7]. This approach applies clinically used drugs to other specific diseases by elucidating new activity and target molecules [8]. This has many advantages, such as no need to test toxicity or evaluate pharmacokinetics [9]. These advantages can considerably reduce the cost and time to develop new drugs, leading to improved success rates [10]. In this study, we adapted the drug repositioning concept to identify candidate drugs to promote differentiation of osteoblast-like cells by inducing Ang1 expression. We selected metformin as such a candidate after screening an FDA-approved drug library with ELISA.

Metformin is currently used as a primary drug for treatment of Type 2 diabetes [11], especially for overweight or obese patients with normal renal function [12]. Metformin improves blood sugar level [13] by activating AMP-activated protein kinase (AMPK) [14] in the liver, thereby inhibiting biosynthesis of fatty acids [15], promoting glucose uptake into cells [16] and inhibiting metabolic syndrome [17]. In this study, we demonstrated that metformin induced osteoblast differentiation and suppressed osteoclast differentiation in an in vitro cell model. Furthermore, metformin prevented femoral head deformity through induction of angiogenesis in an in vivo INFH rat model. Our results provide an effective therapeutic strategy for treatment of INFH via drug repositioning.

## RESULTS

### Metformin increases Ang1 expression and cell mobility in U2OS and MG63 cells

**Figure 1:**
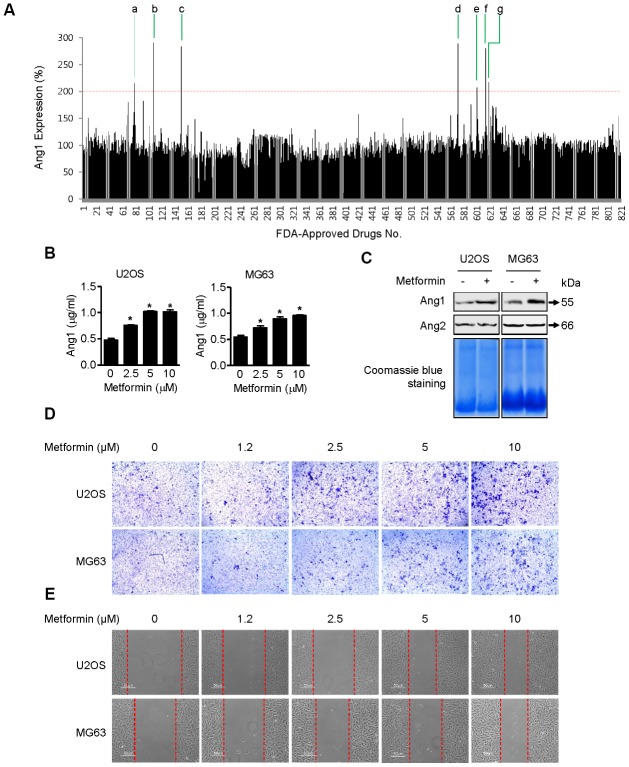
**Induction of Ang1 expression and activation of cell mobility by metformin.** (**A**) Screening result of FDA-approved drugs for Ang1 expression by ELISA. U2OS cells were seeded on 96-well plates. After treating cells with drugs for 1 h, each cell culture medium was transferred to a 96-well plate from the ELISA assay kit. Compounds that induced Ang1 expression over two-fold compared with DMSO vehicle control were selected and are listed in Table 1. (**B**) U2OS and MG63 cells were treated with 2.5, 5, and 10 μM of metformin or an equal volume of DMSO (0.1%) for 1 h. Cell culture medium was collected for Ang1 ELISA. Significant differences between metformin and DMSO control groups are indicated (*P < 0.05, a paired t-test). Experiments were performed in triplicate, and error bars represent standard deviation. (**C**) Cells were treated with 5 μM of metformin or an equal volume of DMSO (0.1%) for 1 h. Cell culture medium from the treated cells was condensed with a Microcon for Western blotting. Coomassie blue staining of SDS-PAGE gels was used for a loading control. Ang1: Angiopoetin1, Ang2: Angipoietin2. (**D**, **E**) (**D**) U2OS and MG63 cells were treated with 2.5, 5, and 10 μM of metformin or an equal volume of DMSO (0.1%) for 6 h. The membrane-traversed cells were fixed and stained with crystal violet solution. (**E**) Cell migration was observed and captured by microscopy at the indicated time point.

Ang1 expression was examined by ELISA in U2OS cells [18] after treatment with a library of 821 FDA-approved drugs (Figure 1A and Table 1), and metformin was selected as one of the strongest candidates. As shown in Figure 1B, metformin increased Ang1 expression in U2OS and MG63 cells. The expression level of Ang1 protein in both cell types was upregulated significantly in a dose-dependent manner in metformin-treated groups. To confirm that Ang1 expression was induced by metformin treatment in U2OS and MG63 cells, total proteins were condensed and analyzed by Western blotting of cell culture media from U2OS and MG63 cells treated with or without metformin. Compared to the DMSO vehicle control, 5 μM of metformin notably increased Ang1 expression in U2OS and MG63 cells. While metformin increased Ang1 expression, angiopoietin2 expression did not differ, implying that metformin is specific for Ang1 expression (Figure 1C). Since proangiogenic Ang1 expression was enhanced by metformin in U2OS and MG63 cells, we tested whether cell mobility (an angiogenic result) of U2OS and MG63 was affected by metformin. As shown in Figure 1D, the membrane-traversed U2OS and MG63 cells were increased by metformin dose-dependently. In addition, the wound healing assay results showed a significant promotion in wound closure following treatment with metformin in U2OS and MG63 cells (Figure 1E).

**Table 1:** Selected drug candidates from the screening for Ang1 expression.

**No.**	**Drugs**	**Position**	**Application**
a	Tolcapone	1-H10	Antiparkinson
b	Metformin·HCl	2-C09	Antidiabetic
c	Sodium Phenylbutyrate	2-G11	Antineoplastic
d	Flucytosine	8-B03	Antimycotic
e	Hydroxyzine Dihydrochloride	8-E02	Antihistamine
f	Lacosamide	8-F05	Anticonvulsant
g	Leucovorin Calcium Pentahydrate	8-F10	Antitoxicity

### Metformin accelerates osteoblast mineralization and upregulates osteoblast differentiation in U2OS and MG63 cells

**Figure 2:**
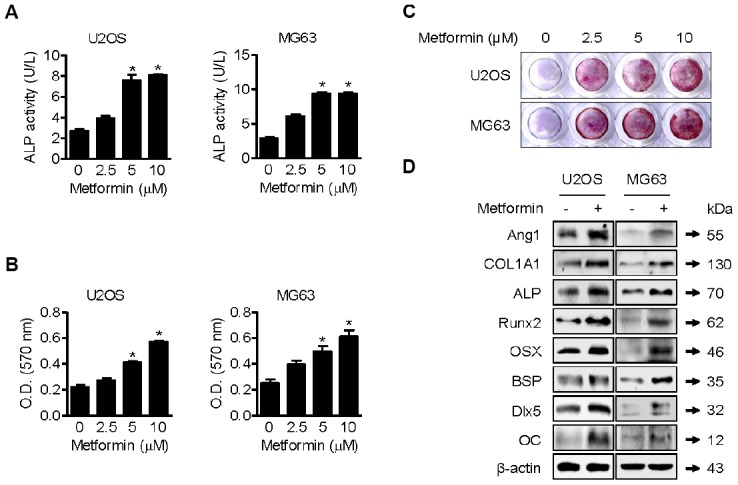
**Promotion of osteoblast mineralization by metformin.** (**A**–**C**) U2OS and MG63 cells were treated with 2.5, 5, and 10 μM of metformin or an equal volume of DMSO (0.1%) for 1 h (**A**) and (**B** and **C**) 20 days. ALP activity in cells was analyzed. Significant differences between metformin and DMSO control groups are indicated (*P < 0.05, a paired t-test). Experiments were performed in triplicate, and the error bars represent standard deviation. (**B**) Cell culture medium containing the proper amount of metformin was changed every other day. The cells were then fixed and stained by Alizarin Red S. (**C**) Alizarin red S-stained cells were extracted, and the optical density was measured with a microplate reader. Significant differences between metformin and DMSO control groups are indicated (*P < 0.05, a paired t-test). Experiments were performed in triplicate, and the error bars represent standard deviation. (**D**) For Western blotting analysis of protein markers of osteoblast differentiation, cells were treated with 5 μM of metformin or an equal volume of DMSO (0.1%) for 1 h. Cells were harvested, and the lysed proteins were resolved on SDS-PAGE and immunoblotted with specific antibodies.

As shown in Figure 2A, ALP activity in metformin-treated (2.5, 5, and 10 μM) U2OS and MG63 cells was significantly higher than in DMSO-treated cells. Osteoblast mineralization refers to deposition of copious amounts of extracellular calcium, which is thought to be essential for bone formation [19]. Metformin treatment for 20 days increased alizarin red S-stained U2OS and MG63 cells in a dose-dependent manner (Figure 2B). Quantification of alizarin red S staining indicated a significant increase in staining after 5 uM metformin treatment compared with DMSO treatment (Figure 2C). These results suggest that metformin enhances osteoblast differentiation and mineralization *in vitro*. As shown in Figure 2D, compared with DMSO vehicle control, 5 μM of metformin notably increased COL1A1, OC, BSP, Dlx5, Runx2, and OSX expression in U2OS and MG63 cells. Moreover, ALP expression level was were dramatically, consistent with the changes in ALP activity.

### Metformin induces phosphorylation of p38

**Figure 3:**
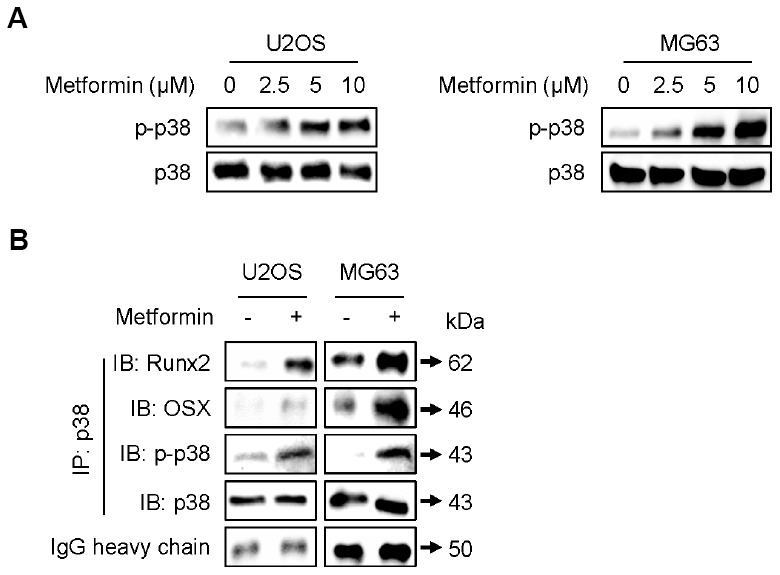
**Increase in phosphorylation of p38 by metformin.** (**A**) Cells were treated with 2.5, 5, and 10 μM of metformin or an equal volume of DMSO (0.1%) for 1 h. Cells were harvested, and the lysed proteins were resolved on SDS-PAGE and immunoblotted with specific antibodies against p-p38 MAPK and p38 MAPK. (**B**) For immunoprecipitation analysis, cells were treated with 5 μM of metformin or an equal volume of DMSO (0.1%) for 1 h. Cells were harvested, and the lysed proteins were immunoprecipitated with antibody against p38 MAPK. The immunoprecipitated proteins were resolved on SDS-PAGE and immunoblotted with specific antibodies against Runx2, OSX, p-p38 MAPK, and p38 MAPK. IgG heavy chain blotting was used as a loading control.

p38 MAPK is reported to interact physically with and transcriptionally activate Runx2 [20] and OSX [21] during osteoblastic differentiation. Thus, we examined whether metformin can induce phosphorylation (activation) of p38 MAPK in U2OS and MG63 cells. As shown in Figure 3A, metformin increased the phosphorylated form of p38 MAPK (p-p38 MAPK) in a dose-dependent manner. Then, we investigated protein interactions between p38 MAPK and Runx2 or OSX after treating U2OS and MG63 cells with metformin. As shown in Figure 3B, p-p38 MAPK expression was much higher and the amount of p38 MAPK protein interacting with Runx2 or OSX was increased by metformin in U2OS and MG63 cells. These results suggest that metformin upregulates transcriptional activity of both Runx2 and OSX proteins via activation of p38 MAPK, leading to high expression of other proteins such as COL1A1, OC, BSP, and Dlx5 for osteoblastic differentiation.

### Metformin inhibits osteoclast differentiation in RAW264.7 cells

**Figure 4:**
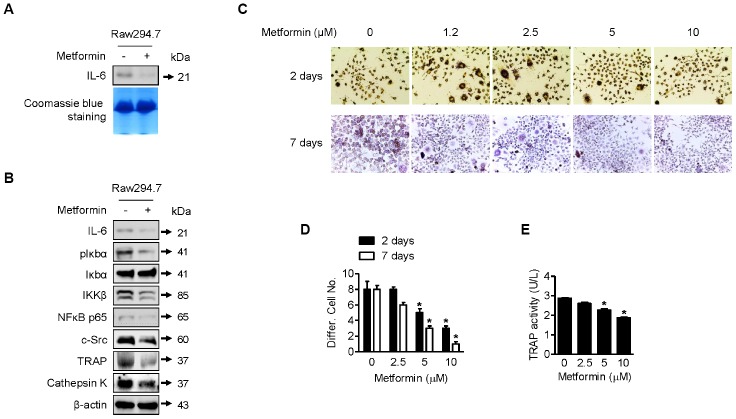
**Downregulation of osteoclast differentiation by metformin.** (**A**) For Western blotting analysis of IL-6 expression by metformin in RAW264.7 cells, cells were treated with 5 μM of metformin or an equal volume of DMSO (0.1%) for 1 h. Cell culture medium from the treated cells was condensed with a Microcon for Western blotting. Coomassie blue staining of SDS-PAGE gels were used for a loading control. (**B**) For Western blotting analysis, cells were treated with 5 μM of metformin or an equal volume of DMSO (0.1%) for 1 h. Cells were harvested, and the lysed proteins were resolved on SDS-PAGE and immunoblotted with specific antibodies against IL-6, pIκbα, Iκbα, IKKβ, NFκB p65, c-Src, TRAP, and Cathepsin K. β-actin blotting was used as a loading control. (**C**) Cells were treated with 2.5, 5, and 10 μM of metformin or an equal volume of DMSO (0.1%) with or without RANKL (0.1 μg/ml) for 2 or 7 days. During incubation, cell culture medium containing the proper amount of metformin was changed every other day. Cells were fixed and stained by the TRAP staining kit according to the manufacturer’s manual. For quantitative comparison, multinucleated cells were counted (**D**). (**E**) RAW264.7 cells were treated with 2.5, 5, and 10 μM of metformin or an equal volume of DMSO (0.1%) for 1 h. TRAP activity in cells was analyzed according to the manufacturer’s protocol. Significant differences between metformin and DMSO control groups are indicated (*P < 0.05, a paired t-test). Experiments were performed in triplicate, and the error bars represent standard deviation.

As shown in Figure 4A, IL-6 expression was decreased by metformin in RAW264.7 cells compared with DMSO control. We next investigated control of IL-6 expression by metformin in RAW264.7 cells in more detail. For this purpose, we performed Western blots of IL-6, p-Iκbα, Iκbα, IKKβ, and NFκB p65. NFκB p65 is a well-known transcription factor that regulates IL-6 expression in osteoclast cells. As shown in Figure 4B, IL-6, p-Iκbα, IKKβ, and NFκB p65 were downregulated by metformin, whereas Iκbα was unchanged. These results imply that NFκB p65 transcriptional activity was reduced through inhibition of IKKβ, leading to the decrease in IL-6 expression. In addition, as shown Figure 4B, expression levels of c-Src, TRAP, and Cathepsin K, well-known protein markers for osteoclast differentiation, were reduced by metformin in RAW264.7 cells. This is consistent with the above IL-6 expression results in terms of preventing differentiation of RAW264.7 cells into osteoclast cells. For measuring TRAP-positive multinucleated RAW264.7 cells, cells were incubated with recombinant RANKL and metformin for 2 or 7 days. As shown in Figure 4C and 4D, metformin decreased TRAP-positive multinucleated cells in a dose-dependent manner that was confirmed by the TRAP activity assay (Figure 4E).

### Metformin protects against ischemic necrosis in the epiphysis of the femoral head

**Figure 5:**
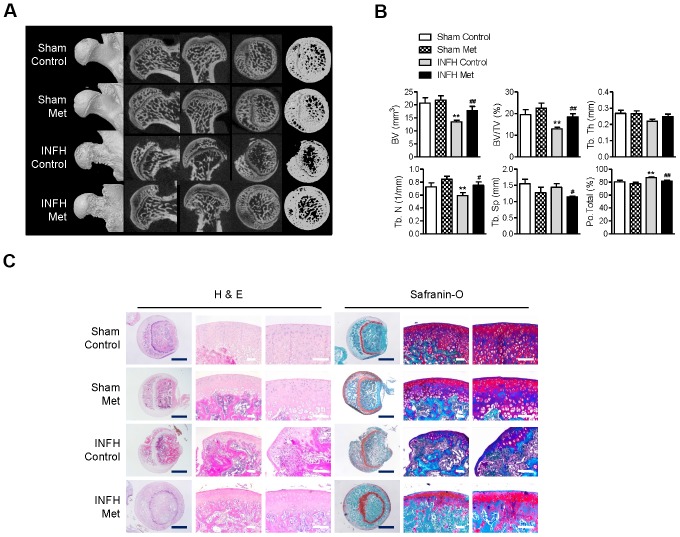
**Protective effect of metformin in the INFH rat model.** (**A**) Representative images of μCT in sham-operated rats with saline injection (Sham Control), sham-operated rats with metformin injection (Sham Met), INFH-operated rats with saline injection (INFH Control), and INFH-operated rats with metformin injection (INFH Met). Images are arranged in order of 3D μCT reconstructions of femoral head area and the coronal, sagittal, and middle areas of femoral heads. (**B**) Morphometric indices in femoral heads of the four groups. Values are presented as the mean ± STD (n = 5). **p < 0.001 versus Sham Control, #p < 0.01 and ##p < 0.001 versus INFH Control. (**C**) Representative images of H & E and Safranin-O staining in the four groups. Black bars = 500 μm, white bars = 50 μm. Abbreviations: BV, bone volume; Tb. N, trabecular number; Tb.Sp, trabecular separation; Tb.Th, trabecular thickness; TV, tissue volume; Po, porosity.

Although there was no significant difference in body weight or general condition between the metformin-injected and control groups, partial absorption of the femoral head epiphysis was observed in the surgery-induced ischemic necrosis of femoral head (INFH) control group (Figure 5A). However, the INFH metformin-injected group showed intact epiphysis of the femoral head, similar to the sham group (Figure 5A). μCT analysis showed decreased bone volume per tissue volume and trabecular number and increased total porosity in the INFH control group compared with the sham group (Figure 5B). On the other hand, bone volume per tissue volume and trabecular number were significantly increased in the INFH with metformin group compared with the INFH control. Moreover, trabecular separation and total porosity were significantly decreased in the INFH with metformin group (Figure 5B). H & E staining and Safranin-O staining showed destruction of the cartilage of the femoral head and accumulation of inflammatory cells in the INFH control. However, the femoral head was intact in INFH with metformin group (Figure 5C). These results suggest that injection of metformin has protective effects against surgery-induced INFH in rats.

### Metformin maintains osteoblast/osteocyte function and vascular density but inhibits osteoclast activity in epiphyseal bone

**Figure 6:**
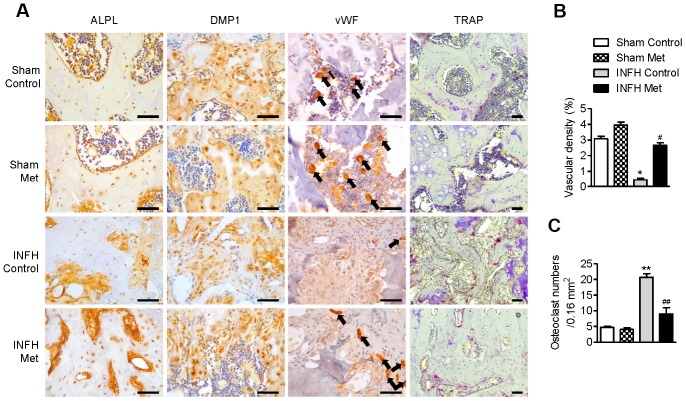
**Working mechanism of metformin in the INFH rat model.** (**A**) Immunohistochemical staining of ALPL, DMP1, vWF, and TRAP in the femoral head epiphysis. Black arrows indicate positive expression of vWF, a marker of endothelial cells of blood vessels. (**B**) Percentage of vascular density in the four groups. Vascular density (%) was calculated as vascular area stained by antibody for vWF-related antigen/total area of each image. Values are presented as the mean ± STD. (n = 4). *p < 0.01 versus Sham Control, #p < 0.01 versus INFH Control. (**C**) Number of TRAP-positive cells per unit area. Values are presented as the mean ± STD (n = 4). **p < 0.001 versus Sham Control, ##p < 0.001 versus INFH Control. Black bars = 50 μm.

To determine the basis of the protective effect of metformin in INFH, we performed immunohistochemical staining in paraffin sections of femoral heads. The expression of ALPL, which is a pre-osteoblast marker, and DMP1, which is a mature osteoblast marker, was reduced in INFH control (Figure 6A). However, the INFH with metformin group showed similar expression of ALPL and DMP1 to the sham group. To evaluate the effects of metformin on femoral head vascularity, femoral heads were immunostained for vWF, a marker of endothelial cells of blood vessels. Immunostaining for vWF antigen revealed more blood vessels in INFH with metformin compared with the INFH control (Figure 6A). Similarly, vascular density was significantly increased in the INFH with metformin group compared with INFH controls (Figure 6B). These results suggest that metformin has a protective role in INFH caused by maintenance of osteoblast function and vascular density. Because of bone destruction in AVN due to osteoclast activity, we evaluated osteoclast activity using TRAP staining. The TRAP-positive cells were increased in INFH controls but decreased in the INFH with metformin group (Figure 6C).

## DISCUSSION

Although there are numerous studies on the anti-angiogenic activity of metformin in cancer models [22–24], we focused on the pro-angiogenic effect of metformin by induction of Ang1 expression in osteoblastic cells. In the same vein as our finding on activation of angiogenesis to overcome ischemic conditions in the femoral head, many publications describe a similar approach using metformin to treat ischemic conditions in the brain [25–27]. Ischemic stroke is a major cause of death and disability. For potential therapeutic strategies, researchers have discovered that chronic administration of metformin prevents ischemic stroke in a mouse model [28]. Its mechanism of action has not been elucidated in detail, but chronic treatment with metformin paradoxically inhibits AMPK in ischemic stroke mouse models. Thus, there seems to be unidentified pleiotropic mechanisms of metformin involved in regulation of other diseases. Recently, Abdelsaid et al*.* showed the potential of metformin to restore angiogenesis in the brain of a rat model with diabetes [29]. They induced ischemic injury in diabetic rats and treated them with metformin. They demonstrated that the excess peroxynitrite formed by ischemia injury in diabetic rats causes brain cell apoptosis in the period after stroke, and metformin prevents this nitrative stress and restores angiogenic function in brain tissue, as evaluated by wound healing and tubule formation assays from primary culture endothelial cells from rats. Thus, the pharmacological functions and mechanisms of metformin via activation of angiogenesis may lead to neuroprotection in cerebral ischemia. As shown in Figure 1D and 1F, we observed dose- and time-dependently increased cell mobility when U2OS and MG63 cells were incubated with metformin. However, it is still not clear whether Ang1 induced by metformin or metformin itself directly affects cell mobility.

Accumulating evidence indicates that patients with diabetes are exposed to higher risk of bone fractures. Although several observational studies have been performed to investigate this relationship, the main risk factors for fracture from diabetes are still not identified [30–32]. A recent meta-analysis study to address the therapeutic effects of seven clinically used anti-diabetic drugs on fractures showed that thiazolidinediones significantly increase fracture risk in older women, but metformin seemed to have no effect on the incidence of bone fracture in patients with diabetes [33].

Metformin, a first-line medication for type 2 diabetes, has been shown to have potential for osteogenic differentiation. A recent study demonstrated that metformin promotes osteogenic differentiation and mineralization of mesenchymal stem cells [34]. That study showed that metformin significantly enhanced ALP activity, mineralized nodule formation, and expression of Runx2 and OSX, consistent with our data (Figure 2A–2C) in an osteoblast cell line. Interestingly, they elucidated the details of the molecular mechanism by examining the effect of metformin on the signaling pathway of LKB1/AMPK, which contains well-established target proteins activated by metformin. Thus, to expand our study, we tested whether metformin affects activation of the LKB1/AMPK signaling pathway in U2OS and MG63 cells. Xinyu et al. reported that, under high-glucose cell culture media, the proliferation rate of MG63 osteoblast cells was lower than under normal-glucose cell culture media, and metformin treatment rescued this suppression of proliferation of MG63 cells [35]. By Western blotting and real-time PCR analysis, they also showed that metformin increases expression of COL1A1, OC, and osteoprotegerin (OPG) with enhanced APL enzyme activity in MG63 cells, also consistent with our current study (Figure 2D).

p38 MAPK has been reported to play a key role in osteoblast differentiation. For example, p38 MAPK is phosphorylated (activated) during osteoblast differentiation in human mesenchymal stem cells [36]. SB203580, a pharmacological inhibitor of p38 MAPK, impairs osteoblast differentiation of MC3T3 cells and downregulates expression of ALP, OC, and COL1A1 [37, 38]. As *in vivo* evidence for activation of p38 MAPK in osteoblast differentiation, several reports have shown that p38 MAPK knock-out transgenic mice have impaired osteoblast differentiation, bone composition, and maintenance [39, 40]. As described in the results section of our study (Figure 3A and 3B), p38 MAPK is phosphorylated by metformin treatment to show stronger binding to Runx2 [20] and OSX [21], which seems to increase their transcriptional activity during osteoblastic differentiation. Previously, Ge et al. demonstrated that p38 MAPK phosphorylates Ser319 of Runx2 using a specific phospho-Runx2 antibody and activates the transcriptional activity of Runx2 according to luciferase assay analysis, leading to osteoblast differentiation [41]. In other reports, p38 MAPK phosphorylated Ser33 and Ser77 of OSX and activated transcriptional activity of OSX via recruitment of p300 and BRG-1 [42, 43]. As shown in Figure 3B, protein interactions between p38 MAPK and Runx2 or OSX were increased with phosphorylation of p38 MAPK by metformin in U2OS and MG63 cells, possibly representing the enhanced transcriptional ability of Runx2 and OSX for regulating other genes related to osteoblast differentiation such as COL1A1, OC, BSP, and Dlx5. At present, we are trying to verify whether Runx2 and OSX bind to and cooperate with each other to increase their protein stability and to locate the cell nucleus for transcriptional activity by metformin. Artigas et al. reported that this interaction requires action of p38 MAPK, and mutant phosphorylation sites of Runx2 and OSX prevented their interaction [44]. However, it is still not clear whether metformin-induced Ang1 or metformin itself directly affects phosphorylation of p38 MAPK with transcriptional activation of Runx2 and OSX. This issue may be resolved using cell lines and mouse models with Ang1 knock-down or knock-out. Considering the number of key actions of p38 MAPK in bone development, we focused on p38 MAPK activation by metformin as a central working mechanism of osteogenesis.

Bone remodeling is the result of a balance between osteoblast and osteoclast differentiation. Thus, a strategy for developing optimal therapies for ischemic osteonecrosis might target either activation of osteoblasts or inhibition of osteoclasts. Osteoclast differentiation is regulated by inflammatory factors and hormones [45]. Among various inflammatory factors, IL-6 has been actively studied and is known to be closely related to osteoclast differentiation [46, 47]. Furthermore, IL-6 may stimulate osteoblast cells to express RANKL to enhance osteoclast differentiation [47]. Therefore, we aimed to investigate IL-6 expression in RAW264.7 osteoclast cells by metformin treatment. As shown in Figure 4A and 4B, IL-6 was less expressed and secreted when RAW264.7 osteoclast cells were incubated with metformin. This result suggests that metformin inhibits osteoclast differentiation via downregulation of IL-6, which was confirmed by other protein markers for osteoclast differentiation and a TRAP assay (Figure 4). Although we showed the NFκB/IKKβ signaling pathway to be involved in regulation of IL-6 expression in RAW264.7 cells by metformin, we need to assess other signaling pathways controlled by IL-6 in osteoclast cells. According to a recent study, IL-6 can activate Janus activated kinase (JAK), leading to phosphorylation of signal transducer and activator of transcription 3 (STAT3) transcription factor, which is responsible for RANKL expression [48]. Thus, we are investigating regulation of the IL-6/JAK/STAT3/RANKL pathway in osteoclast cells with metformin. Hye-Jin et al. demonstrated the anti-arthritis activity of metformin via downregulation of IL-17-producing T cells (Th17) and upregulation of regulatory T cells (Treg) in a collagen-induced arthritis (CIA) mouse model [49]. According to them, metformin administration suppressed osteoclastogenic activity in CIA and expression of genes related to osteoclast differentiation such as RANKL, TRAP, MMP-9, integrin 3, calcitonin receptor, and Cathepsin K, which is consistent with our Western blot analysis (Figure 4B), and osteoclastogenic activity in CIA. These results indicate that metformin is a potential therapeutic option for rheumatoid arthritis and ischemic osteonecrosis.

In our in vivo findings, administration of metformin after ischemic necrosis surgery maintained osteoblast/osteocyte function and vascular density and suppressed osteoclast activity compared to controls. These effects of metformin had a protective role in ischemic osteonecrosis. Our findings supported those of various other in vivo models. First, metformin increased bone healing mediated by Runx2 and AMPK activation after uniform craniotomy in control and diabetic rats [50]. Furthermore, AMPK knockout mice had smaller cortical and trabecular bone mass compared to WT mice [51]. Second, metformin prevented bone loss in ovariectomized rats through regulation of osteoclastogenesis [52, 53]. Third, metformin induced reduction of alveolar bone loss in ligature-induced periodontitis compared to vehicle-treated rats [54]. These data and our results suggest that metformin has a beneficial effect on bone disease. However, high concentration of metformin (200 mg/kg/day) had no effect on bone defects induced by periodontitis or fracture healing [54, 55]. Aurigena et al. suggested that low concentration of metformin (50 mg/kg/day) decreased the inflammatory response, oxidative stress, and bone loss in ligature-induced periodontitis in rats [54]. In addition, very low dose of metformin (10 mg/kg/day) prevented bone loss induced by periodontitis by increasing osteoblast differentiation [56]. Thus, we used low-dose metformin (25 mg/kg/day) in an INFH rat model.

Overall, we demonstrated that metformin increases Ang1 expression and induces bone formation, differentiation, and mineralization in U2OS and MG63 cell lines *in vitro* and increases vascularity and new bone formation in ischemic femoral heads *in vivo* (Figures 5 and 6). This study reveals a new aspect of metformin by providing novel insight into the specific biomarkers that it regulates during osteoblast/osteoclast differentiation. Since metformin is already widely used for patients with diabetes, this research contributes to the expansion of treatment options for ischemic osteonecrosis.

## MATERIALS AND METHODS

### Reagents

Metformin, dimethyl sulfoxide (DMSO), glycerol, glycine, sodium chloride, sodium dodecyl sulfate, Trizma base, and Tween20 were purchased from Sigma (St. Louis, MO, USA). The FDA-approved drug library (SCREEN-WELL® FDA-approved drug library V2) was purchased from Enzo Life Sciences (Farmingdale, NY, USA).

### Cell culture

U2OS, MG63, and RAW264.7 cell lines (ATCC, Manassas, VA, USA) were cultured in Dulbecco's modified Eagle's medium (DMEM; Invitrogen, Carlsbad, CA, USA) with 10% fetal bovine serum (FBS; Invitrogen) and 1% streptomycin/penicillin (Invitrogen) at 37°C in 5% CO_2_.

### ELISA analysis

Based on our previous report [18], Ang1 level in cell supernatant was measured with a sandwich ELISA kit (Abcam, Cambridge, MA, USA).

### Wound healing assay analysis

U2OS and MG63 cells (1 × 10^5^ per well) were seeded in 6-well plates and cultured with serum-free medium 18 h before assay. Then, an artificial wound was scratched into the confluent cells using a P200 pipette tip. Microscopy images (Leica DM IL LED; Leica GmbH, Wetzlar, Germany) were collected immediately for a record of 0 h status, cell culture media was replaced with DMEM supplemented with 1% FBS, and cells were treated with vehicle control or metformin. Migration of cells was observed and captured by microscopy at the indicated time point.

### Migration assay analysis

U2OS and MG63 cells (1 × 10^5^ per well) were seeded in the upper chamber of the chamber migration apparatus (pore size: 8 μm; Corning Life Sciences, Lowell, MA, USA) and cultured with serum-free medium. Then, cells were treated with the vehicle control or metformin in serum-free medium. After 6 h, cells that had traversed the membrane were fixed and stained with crystal violet solution.

### Alkaline phosphatase (ALP) assay analysis

Based on our previous report [18], ALP activity in U2OS and MG63 cells was determined. U2OS and MG63 cells (1 × 10^5^ per well) were seeded in 6-well plates and treated with vehicle control or metformin for 1 h. ALP activity in cells was determined with an ALP activity detection kit (Abcam Inc.). Briefly, the collected cells were washed with PBS, lysed, and centrifuged. The soluble fraction was used for an enzymatic assay. The soluble fraction (80 μL) and 50 μL of 5 mM pNPP substrate were added to 96-well plates and incubated in the dark for 1 h at 25°C. The reaction was stopped by adding 20 μl of stopping solution. The optical density of each sample was measured at 405 nm in a microplate reader (Bio-Rad, Hercules, CA, USA).

### Alizarin Red S staining analysis

Based on our previous report [18], U2OS and MG63 cells were stained and analyzed. U2OS and MG63 cells (1 × 10^4^ per well) were seeded into 96-well plates and treated with vehicle control or metformin for 20 days. The medium was changed every other day, and additional cannabidiol was added with each media change. Cells were fixed on day 20 with 4% paraformaldehyde (Sigma) for 15 min at 20°C. Cells were rinsed twice with deionized water (ddH2O) and reacted with 40 mM Alizarin Red S (Sigma) solution in ddH2O (pH 4.2) for 15 min. Cells were washed twice with ddH2O, and images were captured. For quantitative comparison, stained cells were extracted with 10% (w/v) acetyl pyridinium chloride (Sigma) in ddH2O for 30 min, and the optical density of the samples was measured at 570 nm in a microplate reader (Bio-Rad).

### Western blotting and immunoprecipitation analysis

U2OS and MG63 cells (1 × 10^5^ per well) were seeded in 6-well plates and treated with vehicle control or metformin. Cells were harvested and lysed with RIPA buffer (Cell Signaling Technology, Danvers, MA, USA). Culture media from the treated cells were condensed with a Microcon YM-10 (Millipore, Bedford, MA, USA) for Ang1 Western blotting analysis. Protein concentrations were determined with a Bradford assay (Bio-Rad). Each protein sample was separated on 10 or 12% SDS-polyacrylamide gel and transferred to a nitrocellulose membrane (Millipore). After blocking for 1 h with a buffer containing 0.05% Tween20 and 3% bovine serum albumin (Sigma), the membrane was probed with specific antibodies and incubated overnight at 4°C. Primary antibodies against p-p38 MAPK, p38 MAPK, ALP, COL1A1, BSP, OC (Cell Signaling Technology), RUNX2, OSX, Dlx5, β-actin, IL-6, p-Iκbα, Iκbα, IKKβ, NFκB p65, c-Src, TRAP, Cathepsin K (Santa Cruz Biotechnology, Santa Cruz, CA, USA), Ang1, and Ang2 (Abcam) were used at a 1:1000 dilution. For immunoprecipitation analysis, cells were washed twice with PBS and lysed with RIPA buffer. The lysates were centrifuged and incubated with an antibody, followed by addition of protein A- or protein G-agarose slurry (Santa Cruz Biotechnology). Protein A/G beads were collected, washed, and resolved by 10 or 12% SDS-PAGE and analyzed by Western blotting.

### TRAP staining analysis

RAW264.7 cells (1 × 10^3^ per well) were seeded in 6-well plates. Cells were treated with 100 ng/mL of RANKL (Sigma) and vehicle control or metformin for 2 or 7 days, fixed in 4% paraformaldehyde, and stained for TRAP activity according to the manufacturer's instructions (Abcam). TRAP-positive multinucleated (nuclei>3) cells were scored as osteoclasts using a microscope (Leica DM IL LED; Leica).

### Acid phosphatase (TRAP) activity assay analysis

Acid phosphatase activity of RAW264.7 cells (1 × 10^5^ per well) seeded in 6-well plates was determined with an acid phosphatase activity detection kit (Abcam). Briefly, collected cells were washed with PBS, lysed, and centrifuged. The soluble fraction was used for an enzymatic assay.

### Animals and surgical procedure

The Institutional Animal Care and Ethics Committee of Chonbuk National University approved all experimental procedures in this study (Ethics number: CBNU 2017-0108). We used male Sprague-Dawley rats (10 weeks of age, 320-350 g). All rats were caged individually at 22°C and 50% humidity in controlled rooms having 12-h light/dark cycles. For anesthesia, we administered Zoletil (150 mg/kg) through intraperitoneal injection. We performed ischemic osteonecrosis of femoral head surgery (n=10) and sham surgery (n=10), as previously described [5]. To confirm the effect of metformin on INFH, metformin was dissolved in physiologic saline. During the week after surgery, we administered metformin (25 mg/kg) once a day by intraperitoneal injection to the experimental group, whereas only saline (the vehicle) was injected in the control group. Rats were euthanized by exsanguination under sodium pentobarbital anesthesia 4 weeks after surgery.

### Micro-computed tomography analysis

Four weeks after surgery, we performed micro-computed tomography (μCT) (1076 Skyscan Micro-CT; Skyscan, Kontich, Belgium) and analyzed the findings with CTAn software (Skyscan). All specimens were tested at a constant threshold (~45 %). Femoral bones were analyzed in the center of the femoral neck from the upper margin of the epiphysis of the femoral head.

### Tissue preparation, immunohistochemistry, vascular density measurements, and TRAP staining analysis

Femora dissected from rats were fixed in 10% neutral buffered formalin at 4°C overnight. The specimens were then washed with phosphate-buffered saline and decalcified in 15% EDTA for 2 months, dehydrated, embedded in paraffin, and sectioned at a thickness of 5 μm. We performed staining of slides of sections with hematoxylin and eosin (H&E) or Safranin-O (Sigma) for histological analysis. For immunostaining, sections were deparaffinized, and the EXPOSE Rabbit Specific HRP/DAB Detection IHC kit (Abcam) was used according to the manufacturer’s instructions. The sections were stained using antibodies against ALPL (1:50; Proteintech, Rosemont, IL, USA), DMP1 (1:300; Takara Bio, Shiga, Japan), and vWF (1:100; Millipore, Temecula, CA, USA). Vascular density was evaluated by measuring vWF-related antigen-stained areas, acquired at the most vascularized area of the femoral head from each rat without knowledge of the experimental group. Four images were produced from each of the four rats per group. Vascular densities were measured using an image analysis system (analySIS, Soft Imaging Systems, Germany) and calculated as (vWF-stained vascular area/total image area in ×200 magnification) × 100 (%). To observe osteoclasts, TRAP staining was performed with a TRAP staining kit (Sigma) according to the manufacturer’s instructions.

### Statistical analysis

Data are expressed as mean ± standard deviation of three independent experiments. When the variances were equal, differences between groups were analyzed with a paired t-test in SPSS software (version 19.0; SPSS Inc., Chicago, IL, USA). All statistical tests were two-sided, and P values less than 0.05 were considered statistically significant.
